# The Role of Induced Pluripotent Stem Cells in the Treatment of Stroke

**DOI:** 10.2174/1570159X22666240603084558

**Published:** 2024-06-27

**Authors:** Yasaman Mehdizadeh Darban, Hamid Askari, Maryam Ghasemi-Kasman, Hanie Yavarpour-Bali, Amirabbas Dehpanah, Parnia Gholizade, Nasrin Nosratiyan

**Affiliations:** 1 Student Research Committee, Babol University of Medical Sciences, Babol, Iran;; 2 Cellular and Molecular Biology Research Center, Health Research Institute, Babol University of Medical Sciences, Babol, Iran;; 3 Department of Physiology, School of Medicine, Babol University of Medical Sciences, Babol, Iran

**Keywords:** Stroke, stem cells, induced pluripotent stem cell, transplantation, tumorgenicity, exosomes

## Abstract

Stroke is a neurological disorder with high disability and mortality rates. Almost 80% of stroke cases are ischemic stroke, and the remaining are hemorrhagic stroke. The only approved treatment for ischemic stroke is thrombolysis and/or thrombectomy. However, these treatments cannot sufficiently relieve the disease outcome, and many patients remain disabled even after effective thrombolysis. Therefore, rehabilitative therapies are necessary to induce remodeling in the brain. Currently, stem cell transplantation, especially *via* the use of induced pluripotent stem cells (iPSCs), is considered a promising alternative therapy for stimulating neurogenesis and brain remodeling. iPSCs are generated from somatic cells by specific transcription factors. The biological functions of iPSCs are similar to those of embryonic stem cells (ESCs), including immunomodulation, reduced cerebral blood flow, cerebral edema, and autophagy. Although iPSC therapy plays a promising role in both hemorrhagic and ischemic stroke, its application is associated with certain limitations. Tumor formation, immune rejection, stem cell survival, and migration are some concerns associated with stem cell therapy. Therefore, cell-free therapy as an alternative method can overcome these limitations. This study reviews the therapeutic application of iPSCs in stroke models and the underlying mechanisms and constraints of these cells. Moreover, cell-free therapy using exosomes, apoptotic bodies, and microvesicles as alternative treatments is discussed.

## 1 INTRODUCTION

Stroke is a critical central nervous system (CNS) disorder that is highly associated with mortality and is a result of cancer and myocardial infarction. Despite current therapeutic attempts and better control of stroke risk factors, the average worldwide lifetime risk of stroke is increasing [1]. Moreover, 90% of patients who survive are disabled, which places a significant burden on global healthcare systems [2, 3]. Ischemic and hemorrhagic strokes are the two major types of stroke that often result from clogging and rupturing of brain vessels, respectively [4]. The incidence of ischemic stroke is significantly greater than that of hemorrhagic stroke by 80% [5]. There are two primary certified therapeutic approaches for ischemic stroke: thrombolysis and thrombectomy [6, 7]. However, these treatments cannot sufficiently affect disease outcomes and are contraindicated in different patients Additionally, their application is accompanied by many limitations and complications. Thrombolytic therapy has a very minimal window (within 3-4.5 h after acute stroke onset) and can lead to complications such as hemorrhage problems [8]. Therefore, available therapies are not effective and require novel treatments with broader time frames that are useful for patients with different risk profiles.

The central pathophysiology of stroke is cellular damage and the loss of other nerve cells. Thus, the ideal therapeutic method would be to restore missing neurological functions beyond neurorestorative functions. Physiologically, endogenous regeneration is activated after stroke. Neuronal progenitor cells (NPCs) naturally exist in the Subventricular zone (SVZ) and subgranular zone (SGZ) of the brain [9, 10]. After stroke, certain chemoattractive factors, such as Stromal cell-derived factor-1α (SDF-1α), increase in the damaged area and induce the migration of NPCs to the infarcted area [11-13]. However, due to the inflammation and cytotoxic environment at the injury site, many of these progenitors and newly generated neurons do not survive at the injury site. Thus, the endogenous regenerative process is limited [14]. Despite endogenous regeneration, the use of exogenous stem cells, such as neural progenitors derived from iPSCs, seems to be promising [15].

iPSCs are pluripotent stem cells that can be generated directly from somatic cells and differentiate into any of the three primary germ layers, such as neurons and glial cells [16, 17]. Additionally, there are promising pieces of evidence showing successful transplantation of iPSCs in stroke models, which results in enhanced neurological functions [18]. Unlocking the immense potential of iPSCs has become a focal point in the dynamic landscape of stem cell research. From their inception through forced transcription factor expression in retroviral vectors to the current era of sophisticated methodologies like Sendai viral and Epstein-Barr virus episomal vectors, iPSCs hold the promise of revolutionizing therapeutic applications. This journey, fraught with challenges such as genetic alterations and teratoma formation, underscores the relentless pursuit of safer, more efficient iPSC generation. While recent advancements address concerns about genetic alterations, the method's susceptibility to teratoma formation post-transplantation remains a notable drawback [19].

In this review, we provide an update on the studies using iPSCs in stroke models, describing possible underlying mechanisms and finally explaining the limitations and safety of their usage. Moreover, we discuss cell-free therapy as an alternative cell-based treatment.

## 2 iPSC TECHNOLOGY

In 1962, for the first time, a novel method was discovered in cell reprogramming: the conversion of somatic cells from grown frogs into eggs [20]. Interestingly, Ian Wilmut and his colleague produced dolly sheep from mammalian epithelial cloning cells approximately three decades later [21]. In 2006, iPSCs were derived from somatic cells by Yamanaka and Takasaki. They programmed adult cells into iPSCs by carrying out the expression of four genes, KSOMs (*KLF4*, *SOX2*, *Oct4*, and *c-Myc*), which encode transcription factors [18]. These results proved that even differentiated cells can generate whole tissue. Soon after, research expanded the range of mouse and human iPSCs to include the differences between pluripotent stem cells and iPSCs [18]. In contrast with ESCs, iPSCs have many advantages, including preventing immune rejection [22], reproduction, and simplicity. However, there are many challenges associated with the development of iPSC technology, such as the need for retroviruses or plasmids for generation and their relationship with cancer and genetic instability, as well as increasing the efficiency and maintenance of transgene expression in enough time [23, 24].

Human iPSCs have been utilized in several fields, such as disease modeling. iPSCs are generated from patients and differentiated *in vitro* [25, 26], drug screening [26, 27], and cell treatment improvement [28, 29]. However, additional clinical experiments need to be performed to achieve the standards of efficacy.

Since neurons are so dependent on the use of glucose, blood flow can directly influence them [30]. Both ischemic and hemorrhagic strokes may cause hypoxia in the long term.

Hypoxia hastens the swift exhaustion of ATP, resulting in compromised function of the Na^+^-K^+^ pump. Due to its crucial involvement in neural depolarization, the breakdown of the Na^+^-K^+^ pump hinders neural function, culminating in the liberation of glutamate. Regrettably, neural cells cannot assimilate this surplus of glutamate. Receptors such as N-methyl-D-aspartate receptors (NMDARs) are stimulated by glutamate, thereby enabling extracellular calcium to enter the intracellular compartment [5]. Cell death ultimately occurs due to elevated Ca^2+^ levels, which trigger phospholipase and calpains.

**Fig. (1):**
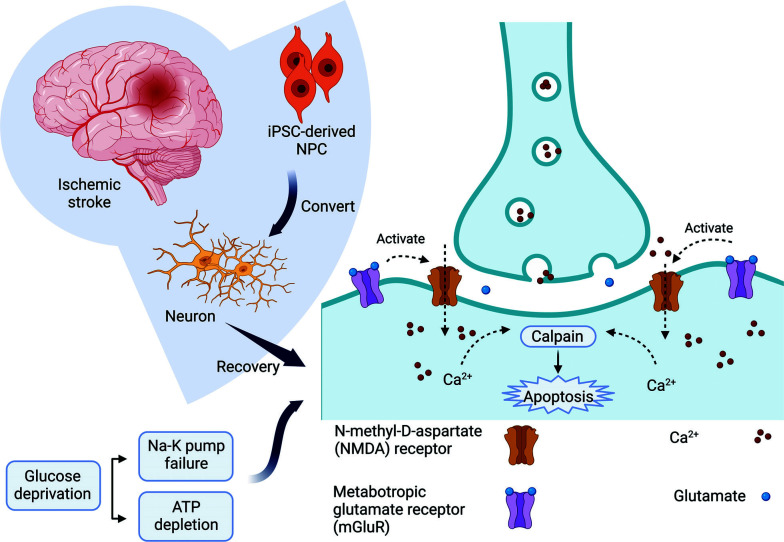
Hypoxia within stroke. Hypoxia damages the Na^+^-K^+^ pump and causes glutamate release, which activates glutamate receptors and leads to calcium flow through the intracellular space. Astrocytes can prevent the process of neural death by accumulating intercellular calcium.

Since neurons are connected by mechanisms such as synaptic transmission, neuromodulation, and paracrine effects, damage affects several cells [31] (Fig. **1**).

## 3 POTENTIAL MECHANISMS IN STROKE RECOVERY

Despite the indication of improved recovery in stroke patients through iPSC implantation, the fundamental mechanisms contributing to this enhancement remain mostly unclear. Recent studies have revealed a potential mechanism by which transplanted NPCs derived from iPSCs may transform during the process after stroke, differentiate into neurons and integrate into the brain [32]. The general outcomes of these studies are as follows:

### 3.1 Neuroprotective Effects

Reductions in the number of apoptotic neural cells and degeneration through the induction of neuronal damage, glial thickness, gliosis, and astroglial scarring are needed to protect neurons after stroke [32].

However, the neuroprotective impacts of transplanting neural stem cells derived from induced pluripotent stem cells (iNSCs) after stroke have been investigated. One research revealed a decreased lesion border in neurons from untreated animals, a characteristic feature of neurodegeneration following stroke. Interestingly, animals treated with iNSCs displayed a level of neuronal survival comparable to that of normal controls, indicating the notable neuroprotective effectiveness of the transplantation of iNSCs. One significant difference was the noted reduction in microglial activation at the lesion border in animals treated with iNSCs, suggesting the potential alleviation of inflammation. Although astroglial activity increased in both groups, treatment with or without iNSCs did not markedly affect astrogliosis. This investigation offers a thorough compilation of evidence that iNSC transplantation holds potential as a neuroprotective intervention. The ability to mitigate neuronal cell death in cortical brain regions after stroke positions iNSC transplantation as a promising avenue for future exploration in the therapeutic realm of neurodegenerative disorders related to stroke [33].

Through a comparative analysis of the transcriptome, 35 secreted factors were identified as overexpressed in iPSC-NPCs, 12 of which are common to iPSCs. Individually, the upregulation of five cytokines (BMP7, CXCL14, FGF8, FGF9, and IGFBP2) that are expressed in iPSC-NPCs provides cortical cell neuroprotection in response to oxygen-glucose deprivation (OGD) and applying these cytokines together recapitulates the observed neuroprotective effects in iPSC-NPCs. The use of neutralizing antibodies against these cytokines leads to the elimination of the benefits observed above, highlighting the crucial role of these factors in the neuroprotective effects of iPSC-NPC grafts.

Facing methyl-phenylpyridinum-induced neurotoxicity, FGF9 prevents dopaminergic neuron destruction [34]. A chemoattractant and dendritic cell activator called CXCL14 [35] has been found, and evidence shows that BRAK/CXCL14 regulates GABAergic synaptic transmission in the adult mouse niche of dentate gyrus stem cells [36]. Moreover, in early postnatal neurogenesis, IGFBP2 has been shown to be overexpressed in the hippocampus [37] and upregulated after murine stroke [38]. Moreover, glioma develops and progresses through IGFBP9 activation [39].

A well-preserved pathway involved in multiple cellular processes, such as neural progenitor cell proliferation, survival of neurons, growth of axons, and formation of synapses, is the Notch1 signaling pathway. Among the 35 cytokines enriched in iPSC-NPCs, BMP2 and GDF11 were identified due to their potential participation in the interaction with Notch1 signaling. The communication between these cytokines and Notch1 signaling might constitute a sophisticated regulatory network that influences the behavior of NPCs and neurodevelopment. This discovery provides opportunities for further exploring the complexity of the molecular mechanisms governing Notch1 signaling and cytokine interactions in the context of NPCs function and neurodevelopmental processes [33].

### 3.2 Inflammation-suppression Capacity

The plasticity of surviving cells in brain tissue increases during the immune response to post-stroke neurogenesis. According to animal studies, pluripotent stem cells can suppress inflammation and increase wound healing [40], which is an important mechanism for promoting functional recovery.

Early iPSC-NPC transplantation leads to a decrease in the production and release of inflammatory cells, including MPO^+^ neutrophils, CD11b^+^ microglia [32], inflammatory cytokines and chemokines in the brain and secondary blood-brain barrier (BBB); interleukins, including IL-1β, IL-6, and tumor necrosis factor-alpha (TNF-α) [32]; and inflammatory signaling proteins, including high mobility group box 1 (HMGB-1), Toll-like receptor 2 (TLR-2), Toll-like receptor 4 (TLR-4), myeloid differentiation primary response 88 (MyD88), p-lx, and nuclear NF-x [41].

In addition, early iPSC-NPC transplantation leads to the modulation of macrophage or microglial responses to stroke [40] and increases the production of anti-inflammatory cytokines, including IL-4 and IL-10 [42]. It also changes the balance of anti- and proinflammatory cytokine signaling. Grafted human iPSC neurons based in the cortex are stimulated by physiological sensory activators. Consequently, afferent synapses are established, and afferent neurons receive direct input from the normal patterns of host brain regions [43].

iPSC-NPC-derived neurons can regenerate neural circuits in the CNS. Modern methods may stimulate or inhibit the activity of grafted cells. This approach helps to determine the main mechanisms of functional recovery in these plants. Upon stroke, iPSC-NPCs begin to produce and multiply neuroblasts, mature and gamma-aminobutyric acid (GABA)-producing neurons of different phenotypes, astrocytes, and oligodendrocytes. The newly produced neuroblasts are transferred to the affected area and differentiate into functional neurons [44].

### 3.3 Restore Cerebral Blood Flow

One of the essential roles of ischemia in brain injury is angiogenesis, which includes a complex and sequential procedure involving the formation of poststroke vessels. These vessels improve tissue perfusion and remodel neurovessels [45], sprouting of axons [46], and remyelination [47]. The evidence also proves the strong communication between angiogenesis and the development of neurological deficits after stroke [47, 48], as shown by the high blood density in vessels, suggesting functional recovery.

Secretion of two important factors, nitric oxide and Vascular endothelial growth factor (VEGF), from iPSC-NSCs has a pivotal role in improving cerebral blood flow in the damaged area [49]. Angiogenesis induction enhances this path [44].

Changes in gene expression are directly related to the recovery of tissues. Endogenous neurogenesis and angiogenesis [50] are both influenced by progenitor cell upregulation, which leads to the production and secretion of trophic factors. The genes involved are the angiogenesis marker Ang1 and brain-derived neurotrophic factor (BDNF) [16]; glial cell-derived neurotrophic factor (GDNF); IL-10; and some growth factors, such as noggin (NOG), neurotrophin3 (NTF3), the neurite branching mediator reticulon4 (RetRtn4) [44], and platelet-derived growth factor receptor beta (PDGFRß) [51].

Another contributor mechanism for angiogenesis is the signal transducer and activator of the transcription 3 (STAT3) pathway. Activated by cellular stimulants such as interleukin 5, interleukin 6, interferon, and epidermal growth factors, this pathway assumes a pivotal role in cellular processes, encompassing cell growth and apoptosis. Extracellular vesicles released by induced mesenchymal stem cells activate the STAT3 pathway, which promotes angiogenesis [52].

In the ischemic area after stroke, SDF-1α, which is related to self-repair, is overexpressed [53]. As a consequence, there is a gradient of SDF-1α expression in the infarct and peri-infarct regions, with robust expression in the core. In conditions resembling ischemia, cells expressing SDF-1α exhibit heightened survival. Secretion of SDF-1α, which binds to the CXCR4 receptor on nearby cells, deactivates cell death mechanisms that occur posttranslationally, restraining Bcl-2-associated death protein (BAD) and leading to the expression of Bcl-2, a prosurvival gene [54]. *In vitro* evidence from the addition of SDF-1α to hydrogen peroxide (H_2_O_2_)-treated cells revealed high expression of Bcl-2 and a decrease in caspase-3-positive cells [55]. SDF-1α protein injected into the ischemic brain notably reduces the size of the infarction and improves motor function due to its role in repair and protection [55].

### 3.4 Reduction in Cerebral Edema

IPSC therapy is crucial in mitigating cerebral edema in rodents subjected to intracerebral hemorrhage (ICH), alongside its anti-inflammatory attributes. The onset of edema, distinguished by unusual fluid retention around hematoma sites and an increasing intracranial pressure, acts as a noteworthy predictor for consequences following ICH, covering primary alongside secondary impairments. The pathogenic routes mesh converge on cerebral edema as a collective outcome [56].

Following ICH, cerebral edema, playing a crucial role in initial and subsequent harm, is prompted by inflammatory reactions. Vasogenic edema is linked closely to inflammatory responses ICH in particular. In rats subjected to iPSC implantation, a decline in inflammatory responses is evident in comparison to the control cohort, implying a plausible mechanism for diminishing edema [32].

The cerebral damage onset subsequent to ICH is associated with inflammatory cells and cytokines intricately. TNF-α, IL-1β, and IL-6 as inflammatory cytokines exert a central influence on both edema and cerebral damage, which aids the enlistment of neutrophils and lymphocytes penetrating to the brain. Such infiltration leads to blood-brain barrier disturbance and additional brain tissue injury. In contrast, IL-10 exhibits promise in mitigating brain damage following ICH [57].

Research outcomes demonstrate a decline in TNF-α, IL-1β, and IL-6, combined with a remarkable rise in IL-10, in the perihematomal tissues of rats subjected to iPSC transplantation. Therefore, iPSC grafts indicate to impede intracerebral infiltration of inflammatory cells and also oversee the generation and cytokines release linked to inflammation in the perihematomal regions of the damaged brain efficiently [32, 57].

**Fig. (2):**
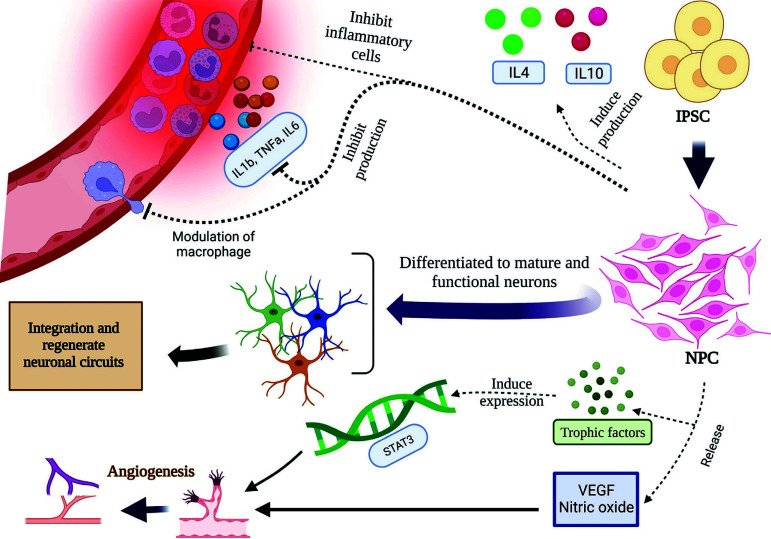
iPSCs enhance neural damage. Engrafted induced pluripotent stem cells (iPSCs) generate neural progenitor cells (NPCs), which subsequently differentiate into functional neurons. Engrafted stem cells induce the production of anti-inflammatory cytokines, including IL-10 and IL-4, and inhibit the production of inflammatory cytokines, such as IL-6, IL-1β, and TNF-α. They also exert their immunomodulatory effect through the inhibition of inflammatory cells such as macrophages, neutrophils, and lymphocytes. Angiogenesis is another mechanism by which stem cells exert their effects. They can induce angiogenesis through the release of vascular endothelial growth factor (VEGF) and nitric oxide. Signal transducer and activator of transcription 3 (STAT3) is another pathway involved in angiogenesis induced by engrafted iPSCs. The STAT3 pathway induces the expression of IL-6, IL-5, interferons, and epidermal growth factors.

The association between cerebral edema and cytokines in stroke is accentuated, wherein IL-1β, IL-6, and TNF-α, in conjunction with the neutrophils and lymphocyte infiltration, play pivotal roles. IPSC treatment mitigates cerebral edema and diminishes the water content of the brain. Additionally, iPSC treatment is proven to oversee cytokines, leading to the secretion of anti-inflammatory cytokines such as IL-10. This is corroborated by research showcasing an elevation in IL-10 levels in animals post-iPSC transplantation [32] (Fig. **2**).

### 3.5 Reduction in Autophagy

Subsequent to brain ischemia, autophagy becomes apparent in diverse cell types, encompassing neurons, oligodendrocytes, and endothelial cells [58]. The autophagy influence on ischemic injury is a matter of dispute, with certain studies accentuating its protective effects while others underscore its harmful consequences [59]. A particular investigation recorded a decline in infarction and improved results in middle cerebral artery occlusion (MCAO) murine models by triggering rapamycin, which is a mammalian target of rapamycin (mTOR) inhibitor inducing autophagy [60]. Research carried out by Shi *et al*. showcased neuronal cell death in relation to autophagy conversely [61]. Notably, attenuating autophagy has exhibited benefits both *in vitro* and *in vivo* for addressing ischemic stroke [62, 63].

Gaining insights into variations in autophagy amid a stroke necessitates an examination of the modulation of protein expression levels. Stroke triggers an elevation in the levels of LC3-II/LC3-I and Beclin-1, coupled with a reduction in the p62 protein. Extracellular vesicles obtained from induced mesenchymal stem cells (iMSC-EVs) offset these modifications by diminishing the levels of LC3-II/LC3-I and Beclin-1, concurrently enhancing p62 protein levels. Moreover, induced mesenchymal stem cell-extracellular vesicles (iMSC-EVs) impede autophagy by triggering the STAT3 pathway and utilizing autophagy inhibitors, such as 3-methyladenine (3-MA) [52].

In the context of ischemic damage, the encouragement of migration and tube formation in human umbilical vein endothelial cells (HUVECs) is facilitated by extracellular vesicles obtained from induced pluripotent stem cell-derived mesenchymal stem cells (iPSC-derived MSC-EVs) [52]. Moreover, these vesicles actively participate in diminishing the damage to the BBB and augmenting its permeability by encouraging proinflammatory chemokines and cytokines after a stroke. When transplanted, neural stem cells derived from human induced pluripotent stem cells (hiPSC-NSCs) are strategically arranged around blood vessels, resulting in a decline in leakage levels [64]. Additionally, the progress in migration and tube formation of HUVECs results in the decrease of the tight junction protein Zonula occludens-1 (ZO-1) and the examination of matrix metalloproteinases, particularly matrix metalloproteinase-2 (MMP-2) and matrix metalloproteinase-9 (MMP-9), both involved in the malfunction of tight junctions among endothelial cells. The transplantation of hiPSC-NSCs effectively diminishes the levels of MMP-9 and the enzyme activity of MMP-9, underscoring their potential to safeguard vascular integrity and mitigating the aftermath of ischemic injury [57].

## 4 SAFETY CONCERNS

As a new treatment method, cell therapy has raised concerns about its efficacy and safety. To reach the clinical stage of the trial, concerns must be proven. The studies on each topic are reviewed below:

### 4.1 Tumor Formation

Overgrowth is a massive concern in cell implantation. Animal studies indicate that intraparenchymal transplantation of iPSCs results in the formation of tumors in MCAO-operated animals before or after surgery [42, 65]. In many studies, there were more tumors in MCAO-operated mice than in control mice, probably due to differences in the microenvironments of their brains [65]. However, many studies have shown that the maturation of iPSCs into iPSC-MSCs, iPSC-NSCs, or other progenies prevents tumor formation [42, 65]. No tumors were observed after four months of injecting human iPSC-derived neuroepithelial-like stem cells into immunocompromised animals [66]. On the other hand, a high proliferation status existed in the early stage of maturation [67]. Therefore, a better mechanism is needed to reach completely differentiated cells to minimize tumor formation risk [68]. Moreover, the administration of viruses such as retroviruses (with retroviral DNA) stimulates tumorigenesis by merging their DNA into host stem cells and influencing the cell cycle [69]. Thus, some studies have attempted non-viral induction, such as the use of an episomal plasmid containing reprogramming factors, and have shown that this approach has become effective [70]. Although the Sendai virus was investigated as a carrier of factors, the infection risk should be considered.

Research has shown an increase in c-myc expression within cylindrical epithelial cells of tumors, and the expression of Neutrophil Gelatinase Associated Lipocalin (NGAL) was detected in cells near tumors [71]. The proto-oncogene c-myc exhibited robust nuclear staining in epithelial cells [72]. Furthermore, c-myc induction occurs in cells that exhibit morphological characteristics of neurons after cerebral ischemia [73], highlighting its crucial role not only in neural cell proliferation and growth but also in promoting vasculogenesis and angiogenesis [74]. For continuous growth, tumors typically necessitate a supply of nutrients and oxygen from nearby vessels through angiogenesis [75]. The significant difference in the expression of c-myc between iPS tumors and the ischemic host brain indicates the tumorigenicity potential of iPCS cells in the ischemic brain compared to that in the sham host brain. Moreover, c-myc contributed to angiogenesis in the tumors of both the Sham+iPS and MCAO+iPS groups, leading to new vessel formation in the tumors, as confirmed by *N*-acetyl glucosamine oligomer (NAGO) staining [71]. Several studies removed both oncogenes and viruses, but recovery without overgrowth was also observed [70]. However, additional investigations are needed, especially in human grafts, because the potential for tumor formation may decrease due to xenograft transplantation. In addition, longer follow-up periods of different methods of induction and differentiation should be investigated in animals [68].

### 4.2 Survival and Migration

The survival rate of implanted cells depends on many factors ranging from the time and site of injection to the number and maturation stage of the injected cells. The survival rate in the ischemic brain is greater than that in the normal brain [65]. However, it still does not exceed 10 percent [44, 62, 63, 68, 76, 77]. Due to the decreased excitotoxicity and inflammatory factor levels and reduced brain edema, studies have shown that the best time for transplantation is one week after stroke when the acute phase has passed, and the environment is more amenable to survival [16].In early to middle developmental stages, the viability of cells is greater than that during full differentiation, and additional studies are needed to fully understand the underlying mechanism involved [40, 77].

Research on transplantation has shown that intracerebral transplantation using biodegradable polyester amide (PEA) 4F4 microspheres (MSs) is effective for treating stroke. Polymeric microparticles, particularly those derived from the biodegradable PEA 4F4, exhibit benefits in terms of easy fabrication, excellent biocompatibility, bioavailability, and controlled delivery of drugs at the local level [78-80]. The incorporation of specific growth factors into PEA 4F4 MS routinely leads to replication of the impact of adding factors during *in vitro* priming. This dual-phase delivery pattern saturates adjacent brain tissue with differentiating molecules following stem cell transplantation. The MS can be loaded with Wnt3A, BMP4, and cyclopamine, which are growth factors recognized for promoting the differentiation of human-induced pluripotent stem cell-derived lt-NESCs into cortical mature neurons [81]. Additionally, research has suggested that the effects of loaded PEA 4F4 MSs on the differentiation of cells are comparable to those routinely used for the addition of these factors during differentiation *in vitro*. Diverse biomaterials have been investigated for their ability to improve NSC viability, proliferation, and differentiation during the transplantation process. Furthermore, MSs made from biodegradable materials based on α-amino acids, specifically PEA 4F4, show no detrimental effects on cell viability *in vitro* and prevent tissue damage upon transplantation into the brains of rats [82]. The observed low to moderate immune response is consistent with earlier findings on the successful implantation of similar PEAs in various applications [83]. The anticipated fate of PEA 4F4 MS degradation products is target tissue absorption or clearance through the blood, liver, or kidney [84]. Evaluating the ideal time for transplantation in stroke patients, data indicate that intracerebral transplantation of loaded PEA 4F4 MS along with neural precursors within a few days after a stroke is likely to yield the most effective results [85]. The controlled release of factors by PEA 4F4 MS over a defined time period offers a promising strategy for augmenting *in vivo* grafted neural precursor differentiation, directing these cells not only toward cortical but also toward other neuronal phenotypes. Such an approach holds the potential to enhance the overall effectiveness of stem cell-based therapies for brain diseases [86].

The quantity of migrated cells is influenced by the relationship between the placement of stem cells and the infarct core [87]. Moreover, contralateral hemisphere injections are more convenient than injections performed ipsilaterally despite the high risk of brain damage [71]. Hippocampal transplantation facilitates the migration of cells and has been shown to cause endogenous neurogenesis [16]. Grafted cells infused within abnormal subdural and epidural spaces eventually disappear within six weeks after transplantation. Additionally, it has been shown that transplanted cell survival is not necessary for outcomes related to brain recovery [33].

New methods have been investigated to increase the survival rate. In another study, preconditioning of hiPS-NPCs with metformin was shown to increase the number of cells that initially survived within a week by improving the proliferation and differentiation rate and reducing immune cell rejection. It also enhances functional motor recovery and reduces the infarct volume [88].

The transplantation of neural progenitors triggers the overproduction of trophic factors, including SDF-1α, in the stroke region [89]. A unique method employs a standalone scaffold, hyaluronan (HA), which has inherent anti-inflammatory properties *in vivo*. This scaffold was utilized for iPSC-NPC transplantation enclosed within a chondroitin sulfate-A hydrogel that interacts with the basic fibroblast growth factor (bFGF). The objective of this approach was to safeguard the transplanted cells in the infarct core [90, 91]. Importantly, HA is capable of stimulating angiogenesis under hypoxic conditions [92]. Furthermore, the hyaluronic acid hydrogel can be tailored with heparin, adhesion peptides, and growth factors for enveloping iPSC-NPCs [93]. On the other hand, fibrin glue, as an alternative biocompatible carrier, has been examined in recent studies as a possibility for improving transplanted cell survival rates. According to the existing body of research, the use of these delivery mechanisms, HA and methylcellulose, has not resulted in substantial improvements [89, 94].

As research continues to unravel the complexities of stem cell transplantation in stroke, these findings contribute to the evolving landscape of regenerative therapies. The utilization of advanced biomaterials, controlled release systems, and innovative preconditioning methods represent a significant stride toward enhancing the efficacy of stem cell-based therapies for brain diseases. Further investigations and clinical trials will be crucial for translating these promising insights into tangible benefits for patients grappling with the aftermath of stroke.

### 4.3 Immune Rejection

In contrast to ESCs, human induced pluripotent stem cell (hiPSCs) transplantation mitigates concerns about immune rejection since hiPSCs can function as autografts. In recent cases, after grafting hiPSCs or their derivatives, the immune system of the host animal was affected by suppressors such as cyclosporine or tacrolimus [66, 87, 93]. Nevertheless, the use of immunosuppressive medications is associated with legitimate concerns about adverse effects and the potential for immune system-related procedures. These concerns include a decrease in immune responses that play a role in post-stroke neurogenesis and angiogenesis, an increase in growth or tumor formation by grafted cells, and a reduction in stem cell migration due to inflammatory signal restriction, which usually leads cells to reach the injury site.

Recently, the focus of various investigations has switched to concentrating on specific strategies for determining the risk of rejection rather than agents involved in immunosuppression. One aspect being investigated involves the production of immunogens such as artificial sialic acid *in vitro* [16]. Allograft transplantation has been the other focus of recent studies. Various studies have shown that the survival rates of ESC-derived neural precursors are comparable despite the use of immunosuppressive agents; therefore, controversy still exists among different observations [16, 32, 44, 49].

The different aspects of hiPSCs are shown in Table **1**, providing opportunities for research on stem cell transplantation [95-103]. This approach addresses various immunological challenges related to the transplantation of hiPSCs and contributes to strategies involving regenerative medicine for neurological diseases.

## 5 CELL-FREE THERAPY

Less than a week after stem cell injection, enhanced function can be found in patients, even though transplantation takes more time [104, 105]. Therefore, in addition to cell replacement, another mechanism exists called the paracrine effect. The paracrine effect is the potential release of nutritional and molecular factors to repair injuries or stroke [106].

Two challenges are predicted through stem cell therapy: the first is tumorigenicity (aforementioned), and the second is the alternative microenvironment *in vitro* rather than *in vivo* [107]. Cell-free therapy is a new approach that seems to be effective at reducing the risk of tumorigenicity, emboli formation, cell rejection, and undesired differentiation and encourages the formation of a microenvironment based on paracrine effects [108-110].

Cellular communication, which is provided by intercellular communication with molecular mechanisms, is one of the key principles of coordination for organ survival. Cell-to-cell contact is a complex intercellular communication in which information is exchanged in the form of membrane vesicles [111]. Extracellular vesicles (EVs) are involved in the paracrine effect of forming a stem cell niche by transmitting molecules that can be sent to target cells [100, 103]. EVs are membrane carriers of proteins, nucleic acids, and lipids. Cell-free therapy relies on stem cells as a source of agents through extracellular signaling pathways. Due to their production mechanism and size, EVs are generally divided into three categories: apoptotic bodies, microvesicles, and exosomes.

Apoptotic bodies are released in the case of cell death. The tumor was 0.8-5.0 μm in diameter and was digested by phagocytes soon after the cargo was released. The apoptotic body is involved in transport and intercellular communication in the CNS [112].

MVs are isolated from the outer cell membrane, and their size can vary within the range of 0.2-0.5 μm. They play a role as mediators of cell-to-cell communications [113]. Hypoxia, shearing stress, an activated cascade, and oxidative injury may increase MV shedding [105, 106]. In addition, MV production is more common in cancer patients and even more common in patients with malignant tumors [104, 107]. Research has shown that MVs are included in the premetastatic niche [114].

### 5.1 Exosomes

Exosomes play a crucial role as paracrine factors of stem cells in cell-to-cell communication and in promoting the microenvironment. It can transfer molecules, such as DNA and RNA. Exosomes are 50 nm to 150 nm in size and are generated by endolysosomes [115, 116]. In contrast with stem cells, exosomes have less immunogenicity and can cross the BBB [117, 118].

Because of the impacts of EVs, such as repairing after stress, removing toxic agents, and increasing tolerance to oxidative stress [119], as well as the potential of crossing the BBB and existing in body fluids, EVs are perfect candidates for transferring therapeutic particles and drugs through the CNS [120]. Stem cell-based therapy, like cell-free therapy, is a promising way to cope with immunogenicity and advance patient abilities in a short period of time. However, additional research is needed to explore the exact mechanisms involving different proteins after their release from EVs.

Various enzymes, such as SOD1 and peroxiredoxin, are released by oligodendrocytes and are subsequently transported by EVs to protect cells from oxidative stress. *In vitro* studies demonstrated that these oligodendrocyte-derived EVs lead to apoptosis and abnormal metabolic activity [121, 122]. Another significant signal worth mentioning is heat shock protein (HSP) transmission along with EVs, which is involved in the process of cellular stress [121]. Additionally, during hypothermia, HSP70 is released through astrocytic EVs [122].

STAT3 is an inhibiting factor affecting autophagy pathways [123]. *In vivo* and *in vitro* studies have shown that STAT3 is deactivated in ischemic stroke. In the peri-infarct areas of rats, the STAT3 pathway was upregulated by iMSC-sEVs poststroke and cultured HUVECs subjected to OGD. Abolishment of the inhibitory effect of iMSC-sEVs on stroke-induced autophagy and angiogenesis partially blocks STAT3 activation. STAT3 reduces the expression of Beclin-1, suppressing autophagy *via* oxidative stress inhibition, and FOXO1 and FOXO3 are autophagy-related signaling molecules [123]. STAT3 may inhibit autophagy by reducing Beclin-1 levels in ischemic stroke patients. The results of the present study demonstrated that STAT3 activation is increased by iMSC-sEVs and is accompanied by a decrease in Beclin-1 expression. However, a STAT3 inhibitor abolished the iMSC-sEV-induced inhibition of Beclin-1. These results indicated that ischemic stroke-provoked autophagy is inhibited by iMSC-sEVs through a STAT3-dependent pathway. The STAT3 signaling pathway is multifaceted in brain vessels poststroke [52].

Utilizing iPSCs in cell-based therapy holds promise as a potential strategy for alleviating ischemic brain injury. Nonetheless, iPSC applications may face challenges due to the inherent tumorigenicity of these cells. Recent studies have attributed stem cells to their paracrine mechanism, with EVs playing an essential role in this process [124, 125]. Technologies involving exosomes derived from stem cells have made significant advancements across various fields [126, 127]. The therapeutic benefits of iPSC-EVs have been demonstrated in various diseases, such as hepatic ischemia-reperfusion injury and acute myocardial ischemia-reperfusion, revealing promising outcomes [128, 129]. However, further research is needed to determine the mechanism of iPSC-EVs in ischemic stroke.

## 6 IPSC DIFFERENTIATION FOR STROKE RECOVERY

### 6.1 iPSCs and Glial Cell Differentiation

Astrocytes are the most common subset of glial iPSCs. They play a role in homeostasis, neuron metabolism, blood flow regulation, and synapse formation [5, 130]. Demyelinating disorders such as white matter stroke (WMS) are characterized by the primary death of glial cells, the loss or dysfunction of oligodendrocytes, and damage to axons and myelin.

iPSCs can differentiate into oligodendrocyte progenitor cells and astrocytes to replace damaged cells after WMS [131, 132].

Treating hiPSC-NPCs with a short exposure to Hypoxia-inducible factors (HIF) activation causes cells to become biased toward permanent differentiation predominantly into prorepair astrocytes. This process allows rapid, efficient, and viable Human-induced pluripotent stem cell-derived glial-enriched progenitor (hiPSC-GEPs) to be produced clinically. Other protocols for the glial differentiation of iPSCs involve long and labor-intensive processes that are inefficient and not well suited for determining the optimal cell number for clinical therapy [133]. hiPSC-GEP transplantation shows promise in white matter stroke repair. These cells migrate widely into the brain, promoting the differentiation of oligodendrocytes and facilitating the recovery of white matter. Unlike localized hiPSC-NPCs, hiPSC-GEPs enhance poststroke motor and cognitive recovery. One study emphasized the impact of hiPSC-GEPs on neural circuitry through connections between brain areas. Notably, ablation of these proteins does not diminish recovery, suggesting that their role lies in inducing local responses and secreting growth factors, distinct from direct tissue repair. This nuanced comparison, considering various hiPSC-derived cell lines, underscores the specificity and potential therapeutic benefits of hiPSC-GEPs [134].

### 6.2 NSCs and iPSCs

Neural stem cells are initially found in the intact brain, especially in the hippocampal dentate gyrus (SGZ) and lateral ventricle (SVZ). NSCs are responsible for neurogenesis and differentiation into neurons, astrocytes, and oligodendrocytes. However, they mostly differentiate into glial cells, which form into glial scars. Glial scars are helpful in healing, but additional neural cells are needed to repair the neural network [135]. Moreover, this potential is limited by the efficiency and low number of possible pathways for migrating NSCs. For instance, the NSCs present in the SVZ and SGZ mostly migrate to the olfactory bulb and granule cell layer, respectively [136]. NSCs contribute to self-renewal in the brain after injury, such as ischemic stroke [137]. Transplanted iPSCs, which are derived from NSCs *in vitro*, can effectively migrate to ischemic lesions chiefly through proinflammatory signals that are increased 24 hours after stroke [17].

Studies have demonstrated that transplantation of iPSC-NSCs into the ischemic brain can improve neurological functions within the first weeks after surgery. These early improvements are mainly a consequence of the anti-inflammatory action of NSCs. NSCs reduce proinflammatory cytokines, BBB permeability, and secondary inflammation after stroke. Additionally, grafted cells raise trophic factors such as BDNF and enable endogenous cells to regenerate and improve angiogenesis and neurogenesis [57]. In addition, in animal trials, no tumorigenicity was indicated after transplanting iPSC-derived NSCs into ischemic mice [49].

Despite these paracrine effects, grafted iPSC-NSCs can directly differentiate into neurons and oligodendrocytes. iPSC-NSCs integrate into the synaptic network of the ischemic brain and receive synaptic inputs from host neurons, similar to the pattern observed in the intact brain. Synergistic functions, as animal models are sensitive to stimulation in the ischemic region. However, differentiated cells appear after the first month after stroke. Therefore, comprehensive surveys are needed to evaluate the efficacy of these treatments on neurological recovery after stroke [17].

### 6.3 Mesenchymal Stem Cells (MSCs) and iPSCs

MSCs effectively alleviate organ defects caused by ischemia and improve behavioral function [42]. Additionally, these cells regenerate neurons in the brain after hemorrhage [138]. Recently, iPSC-MSCs, such as bone marrow-derived MSCs (BM-MSCs), have been used as alternatives to tissue-derived MSCs. Moreover, iPSC-MSCs cause limited cell proliferation, potential rejection, and the requirement for invasive procedures during the uptake of stem cells [68]. The transplantation of iPSC-MSCs into the brains of rats that have experienced hemorrhagic stroke leads to a reduction in hematoma volume, improvement in neurological function for up to two weeks poststroke, and a decrease in inflammatory biomarkers and protein expression associated with oxidative stress, apoptosis, and arterial remodeling [138].

Considering the injection site is crucial. Even when MSCs are injected into one hemisphere, they migrate to the opposite hemisphere intravenously [65]. The restricted differentiation capability of MSCs is offset by enhancing ischemic brain function through the release of diverse factors. These include neuroprotective endothelin, angiogenesis-stimulating VFGF, growth factors such as SDF-1α, and anti-inflammatory factors that downregulate damage-associated protein (DAMP)-related inflammatory signaling [69]. Early-stage dysfunctions can occur due to DAMP-related inflammatory signaling and postinjury secondary inflammation [138].

Despite the recent increase in NPCs, the paracrine effects of MSCs have progressed less strongly toward protecting neurons and astroglial inhibition. NPCs can differentiate into neurons; however, MSCs are a better choice for therapeutic use due to their immunomodulatory effect. Notably, recent studies have shown improvements in behavioral functions following MSC-EV injection after stroke [52].

## 7 CLINICAL TRIALS

The potential of stem cell therapies for addressing brain ischemia has attracted increased attention, as highlighted in a recent meta-analysis by Chen *et al*. [139]. The results emphasized an improvement in neurological functions and quality of life through stem cell therapy, providing promising insights into stroke treatment. Nevertheless, the authors stressed the necessity for additional research to strengthen the evidence supporting the clinical use of stem cell transplantation [139, 140].

Early-stage studies have paved the way for the translation of stem cell-based therapies from preclinical to clinical trials. Since 2005, several clinical studies have investigated the effectiveness of different types of stem cells, such as MSCs [30], magnocellular neurosecretory cells (MNCs) [32, 71], and NSCs [57, 72], in cell-based stroke therapy.

Despite the success of these studies, several challenges still exist. Stem cells, which span pluripotent stem cells (ESCs and iPSCs) to neural and adult stem cells (such as MSCs from various tissues), present potential solutions for treating stroke. Nevertheless, ethical concerns surrounding pluripotent stem cell utilization have led researchers to explore alternatives, such as patient-derived iPSCs. This alleviates worries about immunological tolerance since iPSCs or MSCs lack HLA class II [141].

Despite the promise of these advancements, challenges persist, including uncertainties about the cell extraction efficiency, expansion, differentiation, optimal mode and number of injections. Notably, despite their potential, iPSCs have not yet been included in clinical trials for stroke, primarily due to safety concerns and their early investigation stage. It is essential to address these concerns to establish stem cell therapy as a durable and effective treatment for stroke, signifying the onset of a new era of medical intervention [142].

## CONCLUSION

In conclusion, the emerging domain of stem cell therapy for ischemic stroke represents an equilibrium between potential and obstacles. The diverse nature of safety concerns, including aspects such as tumor development, survival rates, and immune rejection, requires a thorough grasp of the inherent complexities of cell therapy. Addressing crucial concerns, iPSC development into specific lineages has reduced the risk of tumor formation, demonstrating advancements. Nevertheless, challenges persist, necessitating more precise mechanisms for thorough differentiation and careful consideration of the potential implications of viral induction methods.

The crucial survival and migration factors of implanted cells highlight the importance of the injection time, site specificity, and cell maturation stage. Creative methods, as illustrated by the use of biodegradable microspheres for precise local drug delivery, represent substantial progress in tackling these challenges. Furthermore, progress in preconditioning methods and diverse biomaterial investigations has led to the development of new therapies on a stem cell basis.

Worries regarding immune rejection have prompted an hiPSC transition to reduce risks, although inquiries have arisen regarding the utilization of immunosuppressive agents. The investigation of alternative strategies, such as the *in vitro* production of immunogens, highlights the evolution of regenerative medicine, aiming for a delicate equilibrium between rejection and immune-related process risks.

The paradigm shift brought about by cell-free therapy underscores the paracrine effect as a mechanism for improved function following stem cell injection. Extracellular vesicles, especially exosomes, play a pivotal role in this cell-free approach, providing a safer alternative by mitigating the risks associated with cell therapy, such as tumorigenicity and immune rejection.

Although clinical trials exhibit promise in enhancing neurological functions, challenges persist, particularly in translating success observed in preclinical stages to practical clinical applications. Ongoing research and refinement are underscored by ethical considerations and induced pluripotent stem cell safety profiles. With field advancements, advanced biomaterial integration, controlled release systems, and innovative preconditioning methods have the potential to revolutionize stem cell-based therapies as interventions for patients recovering from the aftermath of a stroke.

The transition from laboratory discoveries to clinical efficacy requires ongoing investigation and a careful approach to guarantee the safety and effectiveness of stem cell therapies in treating the intricate landscape of ischemic stroke. Ethical considerations, coupled with a dedication to improving safety profiles, emphasize the ongoing obligation to improve the comprehension and application of our stem cell therapies. While researchers navigate a complex array of challenges, collaborative endeavours within the scientific community play a crucial role in realizing the transformative potential of stem cell interventions for the well-being of patients seeking ischemic stroke recovery.

## Figures and Tables

**Table 1:** Advantages and disadvantages of using iPSCs rather than ESCs/MSCs/NSCs.

**S. No.**	**Advantages**	**Disadvantages and Concerns**
1	Reduced tumorgenicity [49].	Oncogenicity of transcription factors [49].
2	Great source for disease modeling [95].	Inadequate knowledge about optimal time window and cell dose [96].
3	Ability to differentiate into neural cells, NPCs, and vascular endothelial cells *in vitro* [97, 98].	No clinical trial evidence.
4	It can be derived from various kinds of cells [97, 98].	Needs more specific vehicles for transplantation, which is broadly researched [42, 99].
5	Easier methods are available for cell isolation than using bone marrow or embryonic stem cells [100].	Cells take a longer route to become neurons. Therefore, experimental errors become more likely to happen.
6	Reduced possibility of immune rejection after autologous transplantation [101].	Stimulating the inflammatory response because of viral vehicles or vectors [102].
7	Less ethical problems because of autologous implantation [101].	Reprogramming may lead to epigenetic or genetic alterations [103].

## LIST OF ABBREVIATIONS

BAD: Bcl-2-associated Death Protein
BBB: Blood Brain Barrier
BDNF: Brain-derived Neurotrophic Factor
bFGF: Basic Fibroblast Growth Factor
BM-MSCs: Bone Marrow-derived MSCs
CNS: Central Nervous System
DAMP: Damage Associated Protein
ESCs: Embryonic Stem Cells
EVs: Extracellular Vesicles
GABAergic: Gamma-aminobutyric Acid Produced
GDNF: Glial Cell-derived Neurotrophic Factor
H_2_O_2_: Hydrogen Peroxide
HA: Administrated Hyaluronan
HIF: Hypoxia-inducible Factors
hiPSC-NSCs: Human iPSC-derived Neural Stem Cells
hiPSCs: Human Induced Pluripotent Stem Cells
HMGB-1: Inflammatory Signaling Protein Including High Mobility Group Box 1
HSP: Heat Shock Protein
HUVECs: Human Umbilical Vein Endothelial Cells
ICH: Intracerebral Hemorrhage
iMSC-EV: Induced Mesenchymal Cell-extracellular Vesicle
iMSC-EVs: Induced Mesenchymal Stem Cell-extracellular Vesicles
iNSCs: Induced Pluripotent Stem Cell-derived Neural Stem Cells
iPSC-NSCs: Induced Pluripotent Stem Cell-derived Neural Stem Cells
iPSCs: Induced Pluripotent Stem Cells
MCAO: Middle Cerebral Artery Ischemia
MMP-2: Matrix Metalloproteinase-2
MMP-9: Matrix Metalloproteinase-9
MNCs: Magnocellular Neurosecretory Cells
MS: Microspheres
MSC: Mesenchymal Stem Cell
mTOR: Mammalian Target of Rapamycin
MyD88: Myeloid Differentiation Primary Response 88
3-MA: 3-methyladenine
NAGO: *N*-acetyl Glucosamine Oligomer
NGAL: Neutrophil Gelatinase Associated Lipocalin
NMDARs: N-methyl-D-aspartate Receptors
NOG: Growth Factors Noggin
NPCs: Neural Progenitor Cell
NTF3: Neurotrophin3
OGD: Oxygen-glucose Deprivation
PDGFRك: Platelet-derived Growth Factor Receptor Beta
PEA: Poly Ester Amide
RetRtn4: Neurite Branching Mediator Reticulon4
SDF-1*α*: Stromal Cell-derived Factor
SGZ: Subgranular Zone
STAT3: Signal Transducer and Activator of Transcription 3
SVZ: Subventricular Zone
TLR-2: Toll-like Receptor 2
TLR-4: Toll-like Receptor 4
TNF-α: Tumor Necrosis Factor Alpha
VEGF: Vascular Endothelial Growth Factor
WMS: White Matter Stroke
ZO-1: Zonula Occludens-1
